# Disparities in glioblastoma survival by case volume: a nationwide observational study

**DOI:** 10.1007/s11060-020-03428-5

**Published:** 2020-02-14

**Authors:** Rahul Raj, Karri Seppä, Tapio Luostarinen, Nea Malila, Matti Seppälä, Janne Pitkäniemi, Miikka Korja

**Affiliations:** 1 Department of Neurosurgery, University of Helsinki and Helsinki University Hospital, Topeliuksenkatu 5, P.O. Box 266, 00029 Helsinki, Finland; 2 Finnish Cancer Registry, Institute for Statistical and Epidemiological Cancer Research, 00130 Helsinki, Finland; 3 School of Social Sciences, Tampere University, Tampere, Finland; 4 Department of Public Health, School of Medicine, University of Helsinki, Helsinki, Finland

**Keywords:** Glioblastoma, Glioma, Malignant glioma, Epidemiological study, Mortality, Outcome

## Abstract

**Introduction:**

High hospital case volumes are associated with improved treatment outcomes for numerous diseases. We assessed the association between academic non-profit hospital case volume and survival of adult glioblastoma patients.

**Methods:**

From the nationwide Finnish Cancer Registry, we identified all adult (≥ 18 years) patients with histopathological diagnoses of glioblastoma from 2000 to 2013. Five university hospitals (treating all glioblastoma patients in Finland) were classified as high-volume (one hospital), middle-volume (one hospital), and low-volume (three hospitals) based on their annual numbers of cases. We estimated one-year survival rates, estimated median overall survival times, and compared relative excess risk (RER) of death between high, middle, and low-volume hospitals.

**Results:**

A total of 2,045 patients were included. The mean numbers of annually treated patients were 54, 40, and 17 in the high, middle, and low-volume hospitals, respectively. One-year survival rates and median survival times were higher and longer in the high-volume (39%, 9.3 months) and medium-volume (38%, 8.9 months) hospitals than in the low-volume (32%, 7.8 months) hospitals. RER of death was higher in the low-volume hospitals than in the high-volume hospital (RER = 1.19, 95% CI 1.07–1.32, p = 0.002). There was no difference in RER of death between the high-volume and medium-volume hospitals (p = 0.690).

**Conclusion:**

Higher glioblastoma case volumes were associated with improved survival. Future studies should assess whether this association is due to differences in patient-specific factors or treatment quality.

**Electronic supplementary material:**

The online version of this article (10.1007/s11060-020-03428-5) contains supplementary material, which is available to authorized users.

## Introduction

Glioblastoma is the most common malignant brain tumor to be newly diagnosed in adults [1]. The aim of treatment of newly diagnosed glioblastomas is palliative, consisting of maximal surgical resection and, since 2005, consisting of adjuvant concomitant chemoradiation including temozolomide followed by cycles of temozolomide chemotherapy (i.e., the Stupp regimen) [2, 3]. Still, glioblastoma is rare with an incidence of approximately 3/100,000 [4]. Thus, most hospitals see only a few cases every year [5].

In general, hospital volume is frequently associated with various treatment outcomes. Studies of glioblastoma treatment have suggested that survival is improved in high-volume and academic centers [5–9]. However, reliable comparisons between hospitals have been challenging to conduct due to the vast heterogeneity in case-mix between hospitals. Socioeconomic factors and personal insurance, for instance, have a strong influence on glioblastoma treatment and survival in many countries [10]. Given that Finland has the largest human capital [11], one of the highest cancer survival rates [12], and one of the highest health access and quality indexes in the world [13] as well as a tax-funded universal healthcare system, Finland may be considered an optimal country for investigating any volume-outcome associations concerning cancer treatments.

The aim of the present nationwide study was to assess the association between hospital case volume and glioblastoma survival. Due to the reasons mentioned above, and since glioblastoma treatment in Finland has been centralized for decades into five non-profit and relatively similar academic hospitals (with each hospital covering its own population), we believe that any significant effect of case volume on glioblastoma survival is best demonstrated using nationwide Finnish data. Our null hypothesis was that case volume does not influence glioblastoma survival.

## Methods

### Study setting

In Finland, five non-profit academic university hospitals (in Helsinki, Tampere, Turku, Kuopio and Oulu) provide all intracranial surgeries, including glioblastoma resections and biopsies, for their given catchment areas. The Finnish public healthcare system offers equal access to low-cost healthcare. In 2019, for example, the daily fee for short-term institutional care at Helsinki University Hospital was €48.90 with a maximum out-of-pocket payment limit of €683.00 per calendar year. This upper limit included everything from all types of surgery to intensive care unit stays, radiotherapy, and so forth. Furthermore, temozolomide treatment is covered by the Social Insurance Institute KELA with a maximum out-of-pocket payment of €50 per calendar year plus €4.50 for every temozolomide purchase (independent of the amount purchased).

### Finnish Cancer Registry

We have previously described the Finnish Cancer Registry (FCR) in detail [4]. In short, the FCR is a national register that includes all cancers diagnosed since 1953 in Finland. The FCR’s coverage is high, encompassing 96% of solid tumors and 95% of all cases of cancer diagnosed in Finland [14]. The data of the FCR is regularly linked with the Population Register for date of death or emigration and with Statistics Finland for cause of death. Cancer coding follows the nomenclature and coding rules of the International Classification of Diseases for Oncology Third Edition (ICD-O-3).

#### Identification of glioblastoma patients from the FCR

We identified patients aged ≥ 18 years with histopathological (by biopsy or resection) diagnoses of primary glioblastoma using the ICD-O-3 topography codes “C71.0–71.9” and the respective morphology code “9440”. We did not consider morphology codes “9441” (giant cell glioblastoma, N = 25) and “9442” (gliofibroma/gliosarcoma, N = 58). We did not include secondary glioblastomas (previously diagnosed grade II–III gliomas), recurrent glioblastomas, or death-certificate-only (DCO) cases. We have previously shown that the search strategy used in the present study is 97% accurate in identifying glioblastoma cases from the FCR [4].

#### Follow-up

We defined survival time as the amount of time from histopathological diagnosis to death. We followed all patients until death, emigration, or the end of 2015, whichever was earliest. No cases were lost to follow-up.

### Statistical analyses

We divided the five aforementioned academic hospitals into three groups (high-volume, medium-volume, and low-volume) based on the numbers of diagnosed glioblastoma patients in the five catchment areas. This categorization was based upon our previous paper [4].

We calculated age-standardized glioblastoma incidence per 100,000 inhabitants using the European Standard Population 2013 (ESP2013). We reported the incidence rates separately for individuals aged between 18 and 70 years and those aged over 70 years. We also reported the incidence rates separately for the high, medium, and low-volume hospitals. We estimated the relative annual change in the age-standardized incidence rate using a Poisson regression. We used the Davies' test as a significance test to detect changes in the incidence trend [15].

We calculated age-standardized relative survival rates using the Ederer II method, which summarizes patients’ relative excess risk (RER) of death due to cancer by comparing patient survival to that of the reference population (the population of the hospital area stratified by sex, age, and calendar year) [16]. For age standardization, we used the age-group-specific numbers of patients diagnosed in Finland in 2000–2013 as weights in six groups (18–40, 41–50, 51–60, 61–70, 71–80, and 81 years or older at diagnosis).

We compared differences in relative survival between patients diagnosed in the different hospitals by calculating RER using a Poisson regression model for relative survival [17]. This model included seven intervals of follow-up time since diagnosis (0 to < 2 months, 2 to < 6 months, 6 to < 12 months, 12 to < 18 months, 18 to < 24 months, 2 to < 3 years, and 3 to < 5 years), age at diagnosis (the same age groups as in the age standardization), and case volume group. Interaction between follow-up time and age was also included to allow for non-proportional RER of death by age. The first five years of follow-up were considered, and longer survival times were censored at five years. We used the high-case-volume hospital as the point of reference. Thus, an RER under one would indicate that the RER of death is lower than that in the high-case-volume hospital, and conversely, an RER over one would indicate that the RER of death is higher than that in the high-case-volume hospital. We tested differences in RER of death between the hospitals using a likelihood-ratio test. The p values were adjusted for multiple comparisons using the method of Benjamini and Hochberg [18]. Given the distinct differences in survival between patients diagnosed from 2000–2006 and from 2007–2013, as well as the differences in survival between patients under and over 70 years of age [4], these time periods and age groups were analyzed separately. We also described differences in one-year survival rates and median survival lengths (measured in months).

## Results

### Hospitals

**Fig. 1:**
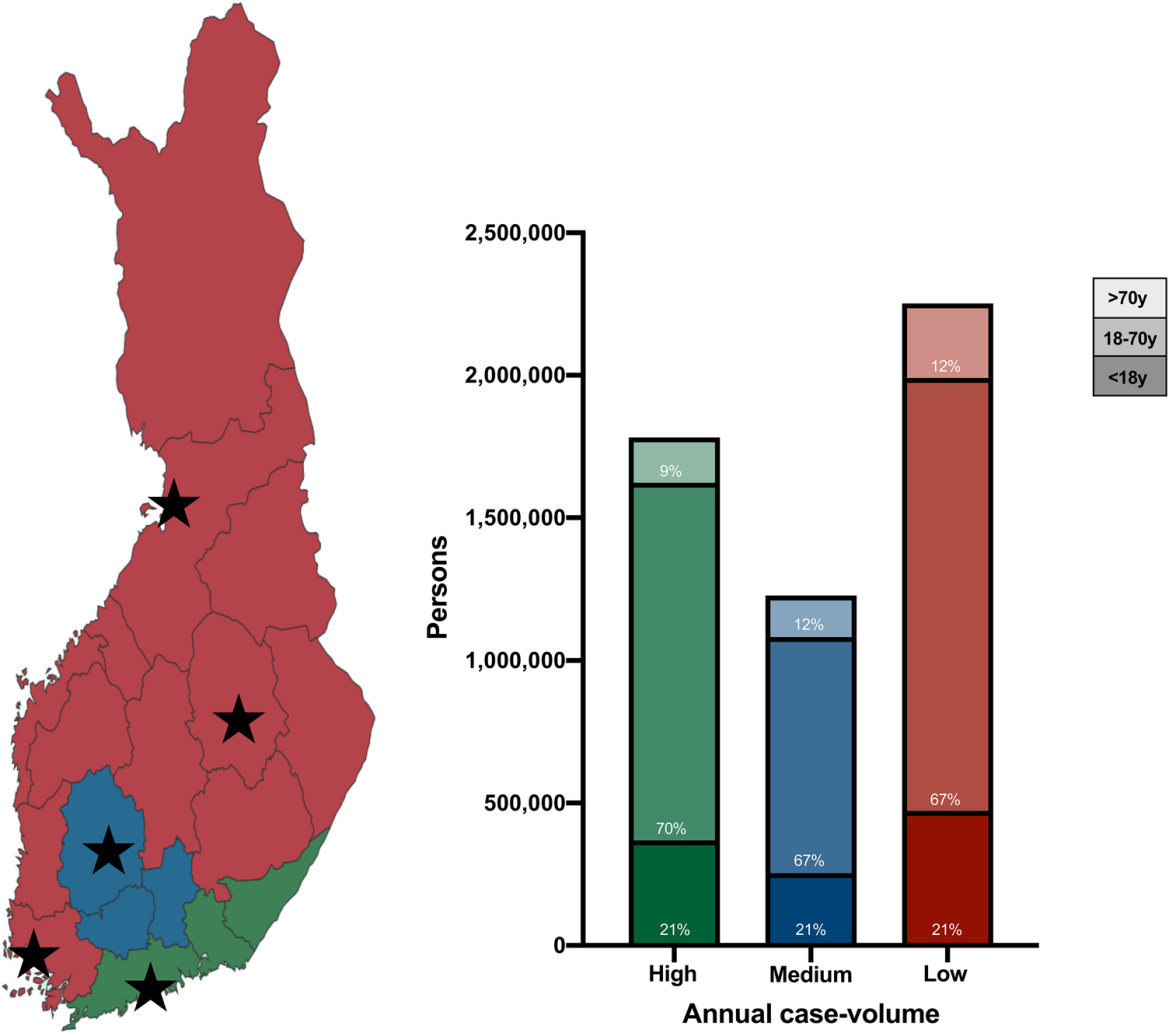
Proportion of persons living in high, medium, and low-volume hospital regions in Finland. Finland’s map to the left: people living in the red areas are being treated in low-volume hospitals, people living in the blue area are being treated in a medium-volume hospital, and people living in the green area are being treated in a high-volume hospital. Stars represent the locations of the five university hospitals. To the right: population distribution according to age in areas covered by a high-volume hospital (green), a medium-volume hospital (blue), and the mean of the low-volume hospitals (red). Number and proportion of persons < 18 years of age (bottom), 18–70 years of age (middle), and > 70 years of age (top)

One hospital was considered to be high-volume (mean annual case volume = 54, total case volume = 761, mean population = 1,782,070), one hospital was considered to be medium-volume (mean annual case volume = 40, total case volume = 560, mean population = 1,228,294), and three hospitals were considered to be low-volume (mean annual case volume = 17, mean total case volume = 241 per hospital, mean population = 750,948, range 701,566–821,218). Of the total Finnish population, 34% lived in a high-volume hospital region, 23% lived in a medium-volume hospital region, and 43% lived in low-volume hospital regions (Fig. 1).

### Patients

We identified 2045 patients with primary glioblastoma diagnosed between 2000 and 2013. The mean and median ages were 62.8 and 63.3 years, respectively. Further, 25% of the patients were older than 70 years of age and 42% were female. There were no noticeable differences in age or sex distribution between the high and medium-volume hospitals. Patients treated in low-volume hospitals were slightly younger and more often male than those in the medium and high-volume hospitals (Table 1).

**Table 1:** Differences in patient characteristics according to case volume

Variable	High-volume (N = 761)	Medium-volume (N = 560)	Low-volume^a^ (N = 724)
Age, year, mean	63.4	62.7	62.3
Age, year, median	63.3	63.2	63.2
≤ 70 year	556 (73%)	416 (74%)	554 (77%)
> 70 year	205 (27%)	144 (26%)	170 (23%)
Sex			
Women	334 (44%)	238 (43%)	282 (39%)
Men	427 (56%)	322 (57%)	442 (61%)
Time of diagnosis			
2000–2006	304 (40%)	262 (47%)	327 (55%)
2007–2013	457 (60%)	298 (53%)	397 (45%)

Categorical variables shown as numbers with percentages. Data from the Finnish Cancer Registry

^a^Sum of all patients treated in three low-volume hospitals

### Incidence

**Fig. 2:**
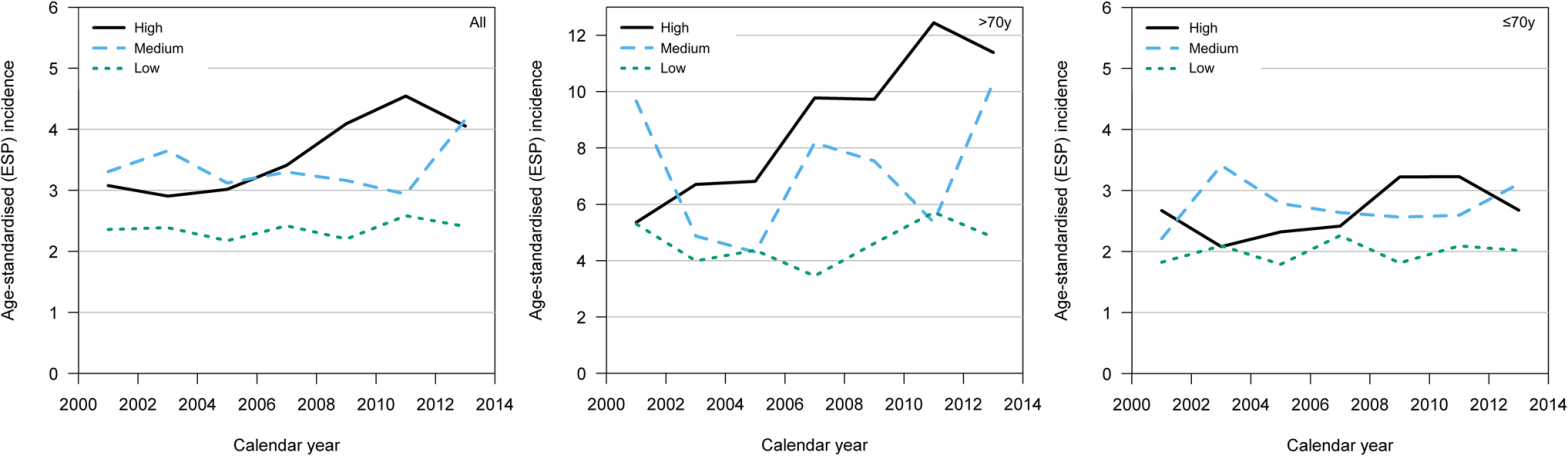
Trends in age-standardized glioblastoma incidence rates from 2000 to 2013 by case volume. From left to right: all age groups, elderly patients (> 70 years of age) and younger patients (≤ 70 years of age). The overall incidence of glioblastoma increased in the high-volume hospital but remained the same in the medium-volume and low-volume hospitals. Still, the incidence of glioblastoma in the medium-volume hospital increased markedly from 2011 onward

The mean age-standardized incidences of glioblastoma for those living in high-volume, median-volume, and low-volume hospital regions were 3.5/100,000, 3.3/100,000, and 2.3/100,000, respectively (Table 2). Throughout the whole study period, the mean annual age-standardized incidence rate of glioblastoma increased in the high-volume hospital region by 3.0% per year (95% CI 1.2–4.9, Davies' test p = 0.33) but did not change in the medium-volume hospital region (0.7%, 95% CI − 1.4 to 2.8 per year, Davies' test p = 0.11) nor in the low-volume hospital regions (0.8%, 95% CI − 1.0 to 2.7 per year, Davies' test p = 0.90; Fig. 2). The increase in the overall incidence of glioblastoma in the high-volume hospital was mainly due to an increase in the incidence of elderly patients (> 70 years of age; Fig. 2).

**Table 2:** Age-standardized incidence rates and incidence rate ratios by case volume

	Age standardized Incidence (95% CI)		
Age group	High-volume	Medium-volume	Low-volume
All	3.5 (3.3–3.8)	3.3 (3.1–3.6)	2.3 (2.2–2.5)
≤ 70 years	2.7 (2.5–2.9)	2.8 (2.5–3.0)	2.0 (1.8–2.2)
> 70 years	9.1 (7.9–10.4)	7.1 (6.0–8.4)	4.6 (4.0–5.4)

Age standardization according to the European Standard Population 2013 per 100,000 people

High-volume hospital used as reference for the incidence rate ratio comparisons. An incidence rate ratio over 1 indicates a higher incidence than the reference

For patients over 70 years of age, the glioblastoma incidence rate was clearly lower in both the medium-volume (7.1 per 100,000) and low-volume (4.6 per 100,000) hospital regions than in the high-volume hospital region (9.1 per 100,000; Table 2). For patients 70 years of age or younger, the glioblastoma incidence rate was lower in the low-volume hospital region (2.0 per 100,000) than in the high-volume (2.7 per 100,000) and medium-volume (2.8 per 100,000) hospital regions.

Within the hospital region, the incidence of glioblastoma increased only in the high-volume region from the period of 2000–2006 to the period of 2007–2013 (for patients ≤ 70 years of age, incidence rate ratio = 1.25, 95% CI 1.05–1.47; for patients > 70 years of age, incidence rate ratio = 1.47, 95% CI 1.10–1.95; Online Resource 2).

### Survival

*The one-year survival rates* in the high-volume, medium-volume, and low-volume hospitals were 39%, 38%, and 32%, respectively. One-year survival rates increased from the period of 2000–2006 to the period of 2007–2013, regardless of case volume (Table 3). From 2007–2013, the one-year survival rates for patients aged ≤ 70 years ranged between 47 and 52%. The one-year survival rates of patients aged > 70 years ranged from 11% in the low-volume hospital to 19% in the medium-volume hospital.

**Table 3:** One-year survival and overall survival according to hospital case volume status

	One-year survival (%, 95% CI)			Overall survival (months, 95% CI)		
	2000–2006	2007–2013	2000–2013	2000–2006	2007–2013	2000–2013
All patients						
High-volume	34 (29–39)	43 (39–47)	39 (36–42)	8.7 (7.6–9.7)	9.9 (9.0–11.1)	9.3 (8.7–10.1)
Medium-volume	34 (28–39)	42 (37–47)	38 (32–42)	7.5 (6.4–8.7)	10.1 (9.0–11.2)	8.9 (8.2–9.8)
Low-volume	27 (22–31)	38 (33–42)	32 (29–35)	6.4 (5.5–7.7)	9.0 (7.8–9.8)	7.8 (7.1–8.6)
Age group ≤ 70 years						
High-volume	42 (36–48)	52 (46–57)	48 (44–52)	10.2 (8.9–11.6)	12.4 (11.0–14.2)	11.4 (10.3–12.3)
Medium-volume	42 (35–49)	50 (43–56)	46 (41–51)	9.4 (7.9–11.4)	11.9 (10.6–13.2)	11.0 (9.9–12.1)
Low-volume	34 (28–39)	47 (41–52)	40 (36–44)	8.6 (7.1–9.8)	11.2 (9.9–12.4)	9.8 (9.1–10.6)
Age group > 70 years						
High-volume	9 (4–17)	17 (11–24)	13 (9–18)	3.8 (2.9–4.8)	4.8 (3.9–5.8)	4.3 (3.6–4.9)
Medium-volume	10 (4–19)	19 (12–28)	16 (10–22)	3.8 (3.1–5.5)	4.3 (3.3–5.4)	3.9 (3.4–5.1)
Low-volume	7 (3–14)	11 (6–18)	10 (6–15)	3.3 (2.4–4.0)	4.0 (3.1–5.4)	3.6 (3.1–5.1)

One-year survival shown as percentages with 95% confidence intervals (CI)

Overall survival shown as medians with 95% CIs

**Fig. 3:**
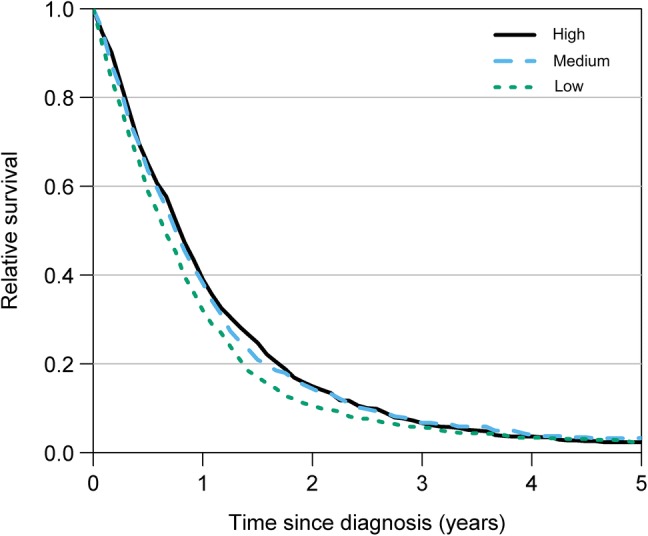
Difference in relative survival between high-volume, medium-volume, and low-volume hospitals (2000–2013). Median overall survival time was longest in the high-volume hospital (9.3 months), followed by that in the medium-volume hospital (8.9 months) and that in the low-volume hospitals (7.8 months)

**Fig. 4:**
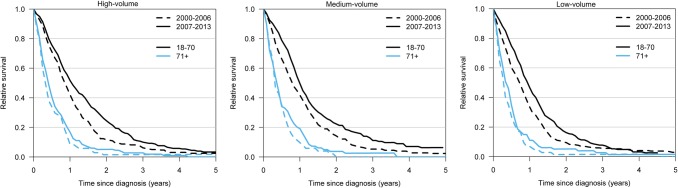
Relative survival by volume status (high to the left, medium in the middle, and low to the right) in the periods of 2000–2006 and 2007–2013, separately for patients aged ≤ 70 and > 70 years

*The overall survival times* ranged from 7.8 months in the low-volume hospital to 9.3 months in the high-volume hospital (Fig. 3). From the period of 2000–2006 to the period of 2007–2013, median survival times increased for all age groups, regardless of case volume (Table 3, Fig. 4). From 2007–2013, the median survival times for ≤ 70-year-old patients ranged between 11.2 months and 12.4 months. For patients aged > 70 years, median survival times ranged from 4.0 months to 4.8 months (Table 3).

*RER of death* was higher in the low-volume hospitals than in the high-volume hospital throughout the study period (Table 4). Throughout the whole study period, RER of death was 19% (95% CI 7–32, p = 0.002) higher in the low-volume hospitals than in the high-volume hospital. For patients aged ≤ 70 years, RER of death was 20% higher (95% CI 6–35, p = 0.016) in the low-volume hospitals than in the high-volume hospital. For patients aged > 70 years, RER of death was 18% higher (95% CI − 5 to 45) in the low-volume hospitals than in the high-volume hospital, but this did not reach statistical significance (p = 0.266). There was no statistically significant difference in RER of death between the high-volume and medium-volume hospitals.

**Table 4:** Relative excess risk of death comparison by case volume status

	High-volume	Medium-volume		Low-volume	
	RER reference	RER (95% CI)	p value*	RER (95% CI)	p value*
2000–2006					
All patients	1.0	1.01 (0.85–1.19)	0.948	1.18 (1.00–1.39)	0.089
≤ 70 years	1.0	0.99 (0.81–1.20)	0.913	1.15 (0.95–1.38)	0.380
> 70 years	1.0	1.06 (0.75–1.49)	0.869	1.30 (0.93–1.82)	0.380
2007–2013					
All patients	1.0	0.99 (0.85–1.16)	0.948	1.19 (1.03–1.37)	0.062
≤ 70 years	1.0	0.97 (0.81–1.17)	0.869	1.22 (1.04–1.44)	0.141
> 70 years	1.0	1.06 (0.79–1.41)	0.869	1.11 (0.85–1.45)	0.869
2000–2013					
All patients	1.0	1.02 (0.91–1.15)	0.690	1.19 (1.07–1.32)	0.002
≤ 70 years	1.0	1.00 (0.88–1.15)	0.952	1.20 (1.06–1.35)	0.016
> 70 years	1.0	1.08 (0.87–1.35)	0.655	1.18 (0.95–1.45)	0.266

Values over one indicates a higher relative risk of death compared to the reference. Values under one indicate a lower relative risk of death compared to the high-volume hospital (reference)

*RER* relative excess risk of death

*p value for the difference to high-volume adjusted for multiple comparisons

## Discussion

In this nationwide study, we found that patients with newly diagnosed glioblastoma had a 19% (7% to 32%) higher RER of death when treated in a low-volume hospital than when treated in a high-volume hospital. Differences in survival, particularly among ≤ 70-year-old glioblastoma patients, seemed to explain this case-volume-associated finding (20%, 95% CI 6–35, higher RER of death when treated in low-volume hospitals vs. high-volume hospitals). We found no difference in survival between the high-volume and medium-volume hospitals. In addition, we found notable differences in the incidence of operated glioblastomas between different case volumes.

A positive correlation between higher hospital case volumes and improved outcomes has been reported for several cardiovascular and oncological procedures [19–21]. Recent studies from the United States have shown improved survival rates for glioblastoma patients treated in centers with more than 23–30 cases per year [5–7]. This finding is in line with our present results, as the mean case volume for the small-volume hospitals was 17 patients, whereas those of the medium and high-volume hospitals were 40 and 54, respectively. Unlike previous studies, our study analyzed RER of death by case volume and case-volume-related survival prior to and after the introduction of the Stupp regimen in 2005 [2]. Perhaps most importantly, we also analyzed case-volume-related survival differences among elderly (> 70-year-old) glioblastoma patients, since they likely comprise the fastest growing group of glioblastoma patients [4]. We did not find a statistically significant association between case volume and survival for elderly patients, whereas a higher case volume was significantly associated with higher survival in younger patients. Still, the RER estimate for elderly patients was similar to the RER estimate for ≤ 70-year-old glioblastoma patients but did not reach statistical significance. This result may have been related to the cohort size resulting in limited statistical power.

In the high-volume hospital region, there was an increase in the incidence of histopathologically confirmed glioblastoma following the publication of the EORTC-NCIC trial [2]. Reasons for this are unclear, but more rapid implementation of new forms of chemotherapy, including temozolomide, may have led to more active histologic confirmation of glioblastoma diagnoses, especially among elderly patients in the high-volume hospital. In other words, when new, promising treatment results were introduced, the results may have been extrapolated as a new standard for elderly glioblastoma patients even though the trial [2] did not include any elderly patients. This means that elderly patients may have been considered as candidates for temozolomide, but only in cases where the diagnosis was histopathologically confirmed. In fact, only 13% of elderly glioblastoma patients received temozolomide in the high-volume hospital in 2005, whereas 33% received temozolomide in 2010 [4]. Thus, differences in the incidence of histopathologically confirmed glioblastoma might reflect a more active diagnostic policy (i.e., lower thresholds for biopsy or resection to obtain histopathological confirmation) for elderly glioblastoma patients in the high-volume hospital. However, lowering treatment thresholds could lead to lower survival rates, presuming that the quality of glioblastoma treatment is similar among different study centers. On the other hand, a more active treatment policy could also cause more patients to undergo tumor resection and actually receive post-operative chemoradiation,this should improve survival [22]. In fact, nearly all elderly glioblastoma patients underwent resections in the high-volume hospital [4], whereas the balance between resections and biopsies in the medium-volume and low-volume hospitals remains unknown. Further studies are needed to pinpoint differences in treatment thresholds and given treatments that may explain the noted differences.

Differences in the incidence of histopathologically diagnosed glioblastoma and survival may also be consequences of differences in other health-related factors, differences in access to care, and differences in true glioblastoma incidence. However, as there are no widely accepted, common risk factors for glioblastoma (except for age), true age-adjusted regional differences in glioblastoma incidence are unlikely. Nevertheless, Finland has a relatively stable gene pool compared to other European countries, with some genetically isolated populations (for example, in Northern and Eastern Finland) [22, 23]. It remains unknown whether there are differences in glioblastoma incidence within the different genetic pools in Finland. However, it is known that health status and life expectancy vary within Finland. For example, individuals living in Northern and Eastern Finland have higher rates of strokes, cardiovascular diseases, mental health issues, and accidents than individuals living in Southern and Western Finland [24]. Thus, since the current median age at the time of glioblastoma diagnosis is approximately 65 years [4], it is possible that people in Northern and Eastern Finland (representing two out of three low-volume centers) die before diagnosis or are due to relevant comorbidities excluded from neurosurgical procedures. As a consequence, regional health and comorbidity differences combined with unit-specific policy variations may affect both the incidence of histopathologically confirmed glioblastoma and disease survival.

In the post-Stupp era (2007–2013), the median survival times were 9.9 months, 10.1 months, and 9.0 months in the high-volume, medium-volume, and low-volume hospitals, respectively. Thus, the survival times in the high-volume and medium-volume hospitals match those of other Nordic countries, while the survival time was slightly shorter in the low-volume hospital. For example, a Norwegian study (time period: 2004–2007, median age = 64 years) found a median survival time of 10.1 months [25] and a Danish study (time period: 2009–2014, median age = 66 years) found a median survival time of 11.2 months [26]. Moreover, studies from the U.S. (Surveillance, Epidemiology, and End Results Program [SEER]) have found median survival times of 15 and 9.7 months [27, 28]. However, it should be noted that only one quarter of the U.S. population is covered by the SEER and that access to neuro-oncological treatment in the U.S. is highly unequal [10]. Thus, due to the major healthcare system differences between the Nordic countries and the U.S., these numbers cannot be directly compared.

Regarding access to care, Finland has a non-profit, government-subsided healthcare system that provides care for all citizens. Still, there are differences in access to primary healthcare that could affect access and delay to glioblastoma care. For example, access to primary care seems to be at its worst in the area of the high-volume hospital in the Helsinki metropolitan area (statistical report 16/2019 THL). Furthermore, delays from the time of referral to the time of access to specialized care is longest in the area of the high-volume hospital (statistical report 25/2019 THL). Treatment delays such as this should decrease survival; thus, it is possible that survival would be even higher in the high-volume hospital if access to care was to improve.

Although it was conducted in a completely different healthcare system, a recent study from the U.S. reported that those glioblastoma patients who travel from low-volume centers to high-volume centers have improved outcomes compared to those who stay in their local, low-volume centers [8]. However, such analyses are biased to some extent by the fact that those who travel are healthier and more often of higher socioeconomic status than those who are not able to travel [29]. In Finland, however, specialized care is centralized according to the geographical location of the patient’s place of residence to one of the five university hospitals. Consequently, receiving specialized elective treatment from a university hospital in another area has only been possible since 2014 and is still exceedingly rare. Thus, it is unclear whether travelling longer distances to a high-volume hospital in a country such as Finland would be beneficial or cost-effective.

Still, there seems to be a difference in survival between hospitals. If this difference is related to lower treatment quality in low-volume hospitals, it is concerning that nearly half (43%) of the Finnish population lives in low-volume hospital regions. However, before obtaining further evidence in support of any of the aforementioned speculations, case-volume-dependent treatment quality differences are a plausible explanation for why both the incidence of operated glioblastomas and survival were highest in the high-volume center. This view of treatment-quality-related disparities is also supported by the results of previous studies [5–8].

## Strengths and limitations

In comparison to previous studies [5–7], our study results are perhaps more generalizable. As mentioned above, the major strength of cancer studies performed in ethnically homogeneous Finland is the fact that Finland has a government-subsided healthcare system, providing affordable, equal care to all citizens regardless of factors such as personal insurance. Thus, the noted differences were unexpected as they were less likely to relate to racial, ethnic, or socioeconomic disparities in the Finnish healthcare setting than in other settings. Furthermore, we have previously validated the FCR with regard to accuracy in identifying glioblastoma [4], making this nationwide register-based study highly reliable. Still, given the rather unique healthcare system in Finland, our results may be less generalizable to countries with more unequal healthcare systems. Moreover, we analyzed the results separately for two age groups and two time periods; this deepened our understanding of the case-volume-dependent disparities. Finally, the fact that the three low-volume hospitals were geographically scattered, further supports the view that the reported differences were likely unrelated to demographic differences.

However, the present study also had some limitations that should be mentioned. First, the FCR does not contain treatment data (e.g. biopsy vs. resection; extent of resection); thus, we were unable to compare treatments in greater detail. Second, the FCR does not contain data regarding adjuvant chemotherapy and radiation therapy. An internal analysis from the high-volume hospital showed that one out of every three elderly patients and nine out of every ten young-to-middle-aged patients received adjuvant radiotherapy with concomitant temozolomide [4]. However, how many patients received adjuvant treatments in the middle-volume and low-volume hospitals remain unclear. Furthermore, differences in geography and travelling distances between the high, middle, and low-volume centers may have affected the actual treatment regimen received by patients following histopathological diagnosis. Third, the FCR did not record tumor genetics during the study period (e.g., O^6^-methylguanine-DNA methyltransferase methylation). Thus, it is possible, albeit unlikely, that differences in tumor genetics between cohorts also played a part in our results. Fourth, we only included patients with histopathological diagnoses of glioblastoma. Thus, our survival and incidence numbers did not include those who died before receiving a histopathological diagnosis (i.e., those not undergoing resection or biopsy).

## Conclusion

We found a significant association between higher case volume and improved survival rates of glioblastoma patients undergoing treatment in academic hospitals. We also found notable differences in the age-adjusted incidences of histopathologically diagnosed glioblastoma within Finland, indicating that diagnostic policies may be more aggressive in high-volume hospitals. Future studies should address not only treatment policy deviations but also access to healthcare and patient pathways (pre and post-clinical diagnosis).

## Electronic supplementary material

Below is the link to the electronic supplementary material.

Supplementary file1 (DOC 87 kb)

Supplementary file2 (DOC 47 kb)
