# Supplementary material for: Disparities in glioblastoma survival by case volume: a nationwide observational study
Source: J Neurooncol. 2020 Feb 14;147(2):361–70. doi: 10.1007/s11060-020-03428-5 (PMC7136186; doi:10.1007/s11060-020-03428-5)
Supplement: Supplementary file 2 — Supplementary file2 (DOC 47 kb) [file 11060_2020_3428_MOESM2_ESM.doc]

| **Online Resource 2:** Age-standardized incidence rates in two time periods and incidence rate ratios between 2000-2006 and 2007-2013 | | | |
| --- | --- | --- | --- |
| **Patient group** | **Age-standardized incidence (95% CI)** | | **Incidence rate ratio** |
| **≤70 years** | **2000-2006** | **2007-2013** | **2000-2006 vs. 2007-2013** |
| High-volume | 2.3 (2.1-2.7) | 3.0 (2.7- 3.3) | 1.25 (1.05–1.47)* |
| Medium-volume | 2.7 (2.4-3.2) | 2.8 (2.4- 3.2) | 1.02 (0.84–1.23) |
| Low-volume | 1.9 (1.7-2.2) | 2.0 (1.8- 2.3) | 1.05 (0.89-1.25) |
| **>70 years** |  |  |  |
| High-volume | 7.1 (5.7-8.9) | 10.8 (9.1-12.8) | 1.47 (1.10–1.95)* |
| Medium-volume | 6.7 (5.2-8.5) | 7.6 (6.1- 9.5) | 1.13 (0.82–1.58) |
| Low-volume | 4.0 (3.2-5.1) | 5.1 (4.2- 6.3) | 1.25 (0.92-1.69) |
| Age-standardized incidences and incidence rate ratios with 95% confidence intervals.  An incidence rate ratio over 1 indicates a higher incidence in 2007-2013 compared to 2000-2006.  **significant 95% confidence intervals (CI)* | | | |
